# The Portuguese long version of the Copenhagen Psychosocial Questionnaire II (COPSOQ II) – a validation study

**DOI:** 10.1186/s12995-017-0170-9

**Published:** 2017-08-09

**Authors:** Susel Rosário, Luís F. Azevedo, João A. Fonseca, Albert Nienhaus, Matthias Nübling, José Torres da Costa

**Affiliations:** 1 Doctoral Programme in Occupational Safety and Health, Faculty of Engineering of the University of Porto, Rua Dr. Roberto Frias, s/n 4200-465 Porto, Portugal; 2 CINTESIS – Centre for Research in Health Technologies and Information Systems and Information and Decision Sciences Department, Faculty of Medicine of the University of Porto, Rua Dr. Plácido da Costa, s/n 4200-450 Porto, Portugal; 3 Department of Health Information and Decision Sciences (CIDES), Faculty of Medicine of the University of Porto, Rua Dr. Plácido da Costa, s/n 4200-450 Porto, Portugal; 4 National Observatory of Pain – NOPain, Faculty of Medicine of the University of Porto, Alameda Prof. Hernâni Monteiro, 4200-319 Porto, Portugal; 5 Faculty of Medicine of the University of Porto, Alameda Prof. Hernâni Monteiro, 4200-319 Porto, Portugal; 6 Allergy Unit, CUF Porto Institute & Hospital, Estrada da Circunvalação 14341, 4100-180; Rua Fonte das Sete Bicas 170, 4460-188 Porto, Portugal; 7 Centre of Excellence for Epidemiology and Health Services Research for Healthcare Professionals (CVcare), University Medical Center Hamburg-Eppendorf, Institute for Health Services Research in Dermatology and Nursing (IVDP), Martinistraβe 52, 20246 Hamburg, Germany; 8 Principles of Prevention and Rehabilitation Department (GPR), Institute for Statutory Accident Insurance and Prevention in the Health and Welfare Services (BGW), Hamburg, Germany; 9 Freiburg Research Centre for Occupational Sciences (FFAW GmbH), Bertoldstr. 63, 79098 Freiburg, Germany; 10 LAETA – Associated Laboratory for Energy, Transport and Aeronautics, Faculty of Engineering of the University of Porto, Rua Dr. Roberto Frias, s/n 4200-465 Porto, Portugal

**Keywords:** Psychosocial risks, Occupational health and safety, Risk assessment (89/391/EEC framework directive), Validation, Portugal

## Abstract

**Background:**

Psychosocial risks are now widely recognised as one of the biggest challenges for occupational safety and health (OSH) and a major public health concern. The aim of this paper is to investigate the Portuguese long version of the Copenhagen Psychosocial Questionnaire II (COPSOQ II), in order to analyse the psychometric properties of the instrument and to validate it.

**Methods:**

The Portuguese COPSOQ II was issued to a total of 745 Portuguese employees from both private and public organisations across several economic sectors at a baseline and then 2 weeks later. Methodological quality appraisal was based on COnsensus-based Standards for the selection of health Measurement INstruments (COSMIN) recommendations. An analysis of the psychometric properties of the long version of COPSOQ II (internal consistency, intraclass correlation coefficient, floor and ceiling effects, response rate, missing values, mean and standard deviation, exploratory factor analysis) was performed to determine the validity and reliability of the instrument.

**Results:**

The COPSOQ II had a response rate of 60.6% (test) and a follow-up response rate of 59.5% (retest). In general, a Cronbach’s alpha of the COPSOQ scales (test and retest) was above the conventional threshold of 0.70. The test-retest reliability estimated by the intraclass correlation coefficient (ICC) showed a higher reliability for most of the scales, above the conventional 0.7, except for eight scales. The proportion of the missing values was less than 1.3%, except for two scales. The average scores and standard deviations showed similar results to the original Danish study, except for eight scales. All of the scales had low floor and ceiling effects, with one exception*.* Overall, the exploratory factor analysis presented good results in 27 scales assuming a reflective measurement model. The hypothesized factor structure under a reflective model was not supported in 14 scales and for some but not all of these scales the explanation may be a formative measurement model.

**Conclusion:**

The Portuguese long version of COPSOQ II is a reliable and valid instrument for assessing psychosocial risks in the workplace. Although the results are good for most of the scales, there are those that should be evaluated in greater depth in future studies. This instrument may contribute to the promotion of a healthy working environment and workforce, providing clear benefits for companies and employees.

**Electronic supplementary material:**

The online version of this article (doi:10.1186/s12995-017-0170-9) contains supplementary material, which is available to authorized users.

## Background

In line with the Europe 2020 objective [1] and the European Union Strategic Framework for Health and Safety at Work 2014–2020 [2], ensuring a healthy and safe working environment contributes considerably to labour productivity and promotes economic growth, competitiveness and welfare [3]. Psychosocial risks are considered the most challenging risk factors across the European Union and a key challenge in modern occupational safety and health (OSH) management, as they are linked not only to health outcomes but also to performance-related outcomes such as absenteeism, ability to work and, in particular, job satisfaction [2, 4]. According to the Framework Directive (89/391/EEC) [5], employers have a legal responsibility to ensure the safety and health of workers in every aspect related to work, including psychosocial risks in the workplace [6].

Although the implementation of these provisions varies from one country to another, the Framework specifies that risks must be identified and assessed, and prevented and managed [7–9]. One of the most important aspects to consider is that risk assessment at work requires the use of valid and reliable methods in order to identify the risk factors in organisations [7, 9–11]. Occupational safety and health legislation therefore places a central focus of risk assessment on preventive approaches [12], which should be considered a priority for organisations [8, 13, 14].

Many measures (mainly questionnaire-based) related to working conditions have been developed, namely the Copenhagen Psychosocial Questionnaire [15, 16], Job Content Questionnaire [17, 18], Effort-Reward Imbalance Questionnaire [19, 20], Pressure Management Indicator [21], Stress Profile [22], Health and Safety Executive Indicator Tool [23], Work Environment Scale [24], General Nordic Questionnaire [25], Job Characteristics Inventory [26], Job Diagnostic Survey [27] and Stress Diagnostic Survey [28], among others, in order to support both employers and employees in the enhancement of OSH processes for prevention and management in organisations [29].

The Copenhagen Psychosocial Questionnaire (COPSOQ) is a comprehensive questionnaire that includes numerous dimensions based on an eclectic set of theories on psychosocial factors at work and on empirical research, rather than being linked to one particular theory [15, 16]. It covers a wide variety of dimensions, describing psychosocial working conditions, and is considered an instrument for research and psychosocial risk prevention in the workplace.

The COPSOQ is an instrument that was developed relatively recently. It was developed in 2000 by Tage S. Kristensen and Vilhelm Borg at the Danish National Research Centre for the Working Environment [15], and revised in 2010 (version II) [16]. In the second version of the Danish COPSOQ study, the psychometric qualities of the instrument were tested in a representative sample of 3517 working Danes between 20 and 59 years of age (52% women, response rate 60.4%). COPSOQ is now one of the most widely used instruments for assessing psychosocial risks in the workplace. It has gained prominent recognition in the scientific community in several countries and has been translated into more than 25 languages, which enables comparison between countries [30, 31]. An increasing number of validation studies have been performed in several countries such as Germany [32, 33], Spain [34], China [35], France [36], Sweden [37], Chile [38] and Iran [39], among others. According to a recent publication by the International Labour Organization [29], the COPSOQ was the first monitoring model to include population-based reference values to assess the need for action and to support the decision-making process concerning preventive measures at the workplace level. Founded in 2009, the COPSOQ International Network (http://www.copsoq-network.org) promotes scientific research and risk assessment using the COPSOQ and aims to facilitate communication between multiple groups. It is therefore linked to governments, universities and research institutions, enterprises and social agents from European and other countries all over the world [40].

The aim of this paper is to present the Portuguese long version of the Copenhagen Psychosocial Questionnaire II (COPSOQ II) and to analyse the psychometric properties of the instrument.

## Methods

The validation study was conducted in two phases. In 2013, the original Danish long version of the Copenhagen Psychosocial Questionnaire II (COPSOQ II) was cross-culturally validated [41, 42] and its appraisal based on COnsensus-based Standards for the selection of health Measurement INstruments (COSMIN) recommendations [43–46]. The Portuguese version showed satisfactory reliability [47, 48]. Secondly, following implementation of the Portuguese version, data was collected between April 2013 and July 2015 and tested for further psychometric quality. Appraisal was based on COSMIN recommendations concerning the psychometric properties of instruments, which are widely accepted internationally. In this validation study, the following COSMIN domains were evaluated: reliability and factorial validity [43–46]. In addition, we compared our results with the original Danish COPSOQ II study.

### Content and structure of the questionnaire

The Portuguese long version of COPSOQ II is a 128-item standardised self-report measure designed for psychosocial risk assessment and prevention. This version has kept the full content and structure of the original Danish long version, in that the 128-item questionnaire consisted of 41 scales reflecting 7 dimensions as outlined in Table 1.

**Table 1:** Domains, scales and number of items in the Portuguese long version of COPSOQ II

Domain	Scale	Number of Items
Demands at work	Quantitative demands	4
	Work pace	3
	Cognitive demands	4
	Emotional demands	4
	Demands for hiding emotions	3
Work organisation and job contents	Influence	4
	Possibilities for development	4
	Variation	2
	Meaning of work	3
	Commitment to the workplace	4
Interpersonal relations and leadership	Predictability	2
	Recognition	3
	Role clarity	3
	Role conflicts	4
	Quality of leadership	4
	Social support from colleagues	3
	Social support from supervisors	3
	Social community at work	3
Work-individual interface	Job insecurity	4
	Job satisfaction	4
	Work-family conflict	4
	Family-work conflict	3
Values in the workplace	Mutual trust between employees	3
	Trust regarding management	4
	Justice	4
	Social inclusiveness	4
Health and well-being	General health perception	1
	Burnout	4
	Stress	4
	Sleeping troubles	4
	Depressive symptoms	4
	Somatic stress	4
	Cognitive stress	4
	Self-efficacy	6
Offensive behaviour	Sexual harassment	1
	Threats of violence	1
	Physical violence	1
	Bullying	1
	Unpleasant teasing	1
	Conflicts and quarrels	1
	Gossip and slander	1
Total	Number of scales 41	
	Number of items	128

Most item responses were scored on a five-point Likert scale with five options: always, often, sometimes, seldom, never/hardly ever or to a very large extent, to a large extent, somewhat, to a small extent, to a very small extent. The following items were reverse-scored: “Do you have enough time for your work tasks?”, “Do you have to do the same thing over and over again?”, “How often do you consider looking for work elsewhere?”, “Do employees withhold information from each other?”, “Do employees withhold information from the management?” and “Does the management withhold important information from employees?”.

The scales were calculated as an average of the scores of the items included and transformed to a range of 0 to 100, with high values representing a high level of the concept being measured. The long version of COPSOQ II also includes questions aimed at the sociodemographic characterisation of the participant. The questionnaire takes 30 min to complete. To score the COPSOQ II scales, at least half of the items should be answered for calculating a particular scale [16]. The Portuguese questionnaire is freely available in the public domain as a PDF download from http://www.copsoq.pt/ [49].

### Study sample

The study was conducted in 34 companies located in the north and centre of Portugal, between 1 April 2013 and 31 July 2015. It was approved by the Ethics Committee of the University of Porto. After being properly informed about the aim of the study, all of the participants signed the consent form prior to being issued with the questionnaire.

**Fig. 1:**
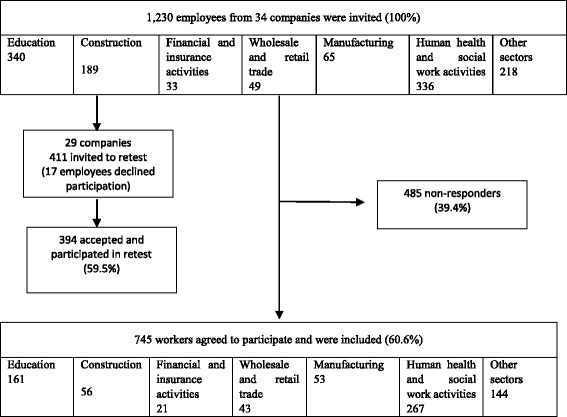
Flow chart showing participation according to the Classification of Economic Activities in the European Union NACE – Rev. 2

The sample included a total of 745 employees from both private and public organisations across several economic sectors (education, construction, wholesale and retail trade, financial and insurance, manufacturing, human health and social work, other sectors) at the baseline assessment (*N* = 745). A retest was conducted after two weeks (7–17 days) to assess reproducibility (*N* = 394). Figure 1 provides details of the participants according to the Classification of Economic Activities. For the current study, we included all workers aged 18 to 65 who were willing to participate in the study and who gave their informed consent.

The response rate was 60.6%. For test-retest validation, the response rate was 59.5%. The sample size included in this study was based not only on COSMIN recommendations (excellent sample size: ≥ 100), [44] but also on the recommendations of Comrey and Lee [50] and McCullum et al. [51], who recommend more than 640 participants for factor analysis (in this case, based on the number of subjects per item/variable: 5 * 128 = 640), as well as in accordance with the recommendations of the Ethics Committee of the University of Porto.

The sample was classified by different sectors of economic activity according to the nomenclature of the Portuguese Classification of Economic Activities (CAE) Revision 3 (CAE – Rev. 3) [52], which is harmonised with the Classification of Economic Activities in the European Union (NACE – Rev. 2) [53] and the International Standard Classification of Activities, Revision 4 (ISIC – Rev. 4) of the United Nations [54]. The classification used data from Pordata, the Data Base of Contemporary Portugal [55] (Additional file 1).

Overall, the “education” and the “human health and social work activities” sectors of economic activities in our sample are considerably higher than in the general working population. The “construction” and “financial and insurance activities” displayed values very close to the population. Furthermore, the “Other sectors” that were considered (E, J, M, N, O, R, S, T, U), despite covering nine more sectors than expected, also displayed very close values. However, the “wholesale and retail trade” and “manufacturing” sectors of our study show representative values far below those for the population in general, suggesting that they should be confirmed by an appropriate sample in future. The characteristics of the study are shown in Tables 2 and 3. The majority of the participants were female (65.6%). The average age of the respondents was 39 (SD = 9.9), with a range of between 19 and 65. The distribution of organisations between public (*n* = 300) and private (*n* = 445) sectors was nearly balanced. Professional groups were classified according to the Portuguese Classification of Occupations [56] and are shown in Table 3. The 2010 Portuguese Classification of Occupations is the most recent international framework (according to the International Standard Classification of Occupations – ISCO 2008) [57].

**Table 2:** Characteristics of the study population

	n	%
Total participants	745	
*Gender*		
Female	489	65.6
Male	256	34.4
*Age distribution*		
19–29	141	19.0
30–39	261	35.0
40–49	194	26.0
50–59	139	18.7
60–65	10	1.3
*Marital status*		
Single	241	32.3
Married	376	50.5
Cohabiting	62	8.3
Divorced	54	7.2
Widowed	12	1.6
*Education*		
≤ 9th year	100	13.4
10th to 12th year	177	23.8
Bachelor	20	2.7
University degree	318	42.7
Postgraduate degree	1	0.1
Master’s degree	102	13.7
PhD	27	3.6
*Economic activities*		
Manufacturing	53	7.1
Construction	56	7.5
Wholesale and retail trade	43	5.8
Financial and insurance activities	21	2.8
Education	161	21.6
Human health and social work activities	267	35.8
Other sectors	144	19.3
*Sectors*		
Public	300	40.3
Private	445	59.7

**Table 3:** Distribution of professionals groups

Occupation CNP ^a^	n	%
Management of companies and public administration	18	2.4
Technical and scientific professionals and intellectuals	341	45.8
Technical and associate professionals	113	15.2
Administrative employees	105	14.1
Workers in catering services, personnel, security, etc.	122	16.4
Skilled agricultural and fishery	0	0
Tradespeople and skilled workers in manufacturing	2	0.3
Plant and machine operators, assemblers	29	3.9
Unskilled workers	14	1.9
Missing value	1	0.1
Total	745	100

^a^ Portuguese National Classification of Occupations

### Study procedure

The procedure was initiated by presenting the study to organisations across several sectors of economic activity. The organisations that were contacted and were available to participate in the study formalised their interest with a signed consent. In every organisation, we tried to cover employees belonging to different hierarchical levels and in different functions in order to ensure that the sample was representative. Data collection activities were developed according to the way each institution worked and in accordance with the dates stipulated in the study.

Data collection included questionnaires available in paper format or as a digital survey. Of the 34 companies in total, digital survey data was collected in three. Before taking part in the digital survey, participants had to meet the following criteria: aged 18 to 65, with each participant having a computer permanently assigned to them for the performance of their duties and willing to participate in the study and to give their informed consent. The COPSOQ II paper format was used and completed in convenient rooms on the organisations’ own premises. The questionnaires were delivered directly to the participants who were supervised while they completed the questionnaire. In the case of the digital survey, the participants filled in an online consent form and completed the online questionnaire. The online questionnaire was made available in order to facilitate data collection, and employees received an email invitation encouraging them to fill out the form at a time and place of their choosing. Employees had 3 weeks to complete the survey and non-respondents received two email reminders during this time. For test-retest validation, similar data collection (paper format or online survey) was conducted after 2 weeks to assess reproducibility. All of the organisations have received a report with a summary of their results.

### Psychometric and statistical analysis

Data analysis was performed and included descriptive statistics using mean and standard deviation (SD). The assessment of the psychometric validity of the Portuguese version of the COPSOQ II followed the COSMIN recommendations [43–46] as well as internationally recommended standards [58–61], and included:

(i) the internal consistency of the 41 scales (test and retest) through Cronbach’s alpha;
(ii) test-retest reliability within two weeks was estimated by the intraclass correlation coefficient (ICC) for quantitative variables;
(iii) descriptive statistics comprising mean and standard deviation for all scales;
(iv) floor and ceiling effects;
(v) response rate (test) and follow-up response rate (retest);
(vi) missing values; and
(vii) exploratory factor analysis.

The items in COPSOQ II were analysed using explorative factor analyses within each of the seven major domains: *Demands at work; Work organisation and job content; Interpersonal relations and leadership; Work-individual interface; Values at the workplace; Health and well-being* and *Offensive behaviour*.

The assessments of internal consistency and test-retest reliability were performed according to available recommendations [58, 59]. Analysis of internal consistency was undertaken by assessing Cronbach’s alpha. As recommended by Nunnally and Bernstein [60], a Cronbach’s alpha of 0.70 is the threshold value for this assessment. The original Danish study [16] also considered the conventional threshold of 0.70.

For the interpretation of the magnitude of the intraclass correlation coefficient (ICC), an ICC greater than 0.70 was considered adequate [62, 63].

A descriptive statistics (mean and standard deviation) analysis was performed for sociodemographic data and for all 41 scales.

Similar to the original Danish COPSOQ II study, floor and ceiling effects, defined as the proportion of respondents selecting the lowest (floor) and highest (ceiling) response options for all items in a scale, were determined for all scales.

The missing values considered if respondents had answered less than half of the questions in a particular scale, and was analysed for all 41 scales.

Exploratory factor analysis was conducted following a recommendation by the Ethics Committee of the University of Porto. Factorial validity was assessed by definition and evaluation of the factor structure of the instrument using methods of exploratory factor analysis [59, 64, 65]. Models of exploratory factor analysis were defined using principal components analysis for factor extraction [59, 64, 65]. The extraction of the main factors was performed using varimax rotation with Kaiser normalisation. Selection of the number of factors to retain took into account Kaiser’s criterion (eigenvalues greater than one); graphical analysis of the scree plot; a criterion based on the total variance explained (at least greater than 50%); and the Kaiser-Meyer-Olkin (KMO). In the factor analysis, the missing items were handled by using the list-wise deletion method [66]. For all hypothesis tests, a significance level of α = 5% was used. Statistical analyses were performed using the Statistical Package for the Social Sciences (SPSS) v20.0® software program.

## Results

A total of 745 employees from 34 companies completed the questionnaire. The average age of the participants was 39 (SD = 9.6). The majority (65.6%) of respondents were female. The participants worked an average of 42.9 h/week (SD = 7.2) and had been in their current jobs for 9.4 years (SD = 9.5) on average. The rate of participation in the test (*N* = 745) was 60.6%, and in the retest (*N* = 394) it was 59.5%. The scale characteristics for the dimensions in COPSOQ II are shown in Table 4.

**Table 4:** Comparison of the reliability and summary descriptive statistics between the Portuguese (*n* = 745) and the original COPSOQ II Danish (*n* = 3517) study sample

Domain	Scale	Danish	Portuguese		Danish		Portuguese		Danish		Portuguese		Danish	Portuguese	Portuguese
		Cronbach’s α *n* = 3517	Cronbach’s α Test *n* = 745	Cronbach’s α Retest *n* = 394	Mean	SD	Mean	SD	% Floor	% Ceiling	% Floor	% Ceiling	Missing (%)	Missing N (%)	Test-retest reliability ICC (95% CI)
Demands at work	Quantitative demands	0.82	0.69	0.67	40.2	20.5	36.3	18.2	2.9	0.3	3.6	0.3	2.2	2 (0.3)	0.818 (0.770–0.859)
	Work pace	0.84	0.74	0.72	59.5	19.1	63.1	19.2	0.5	3.4	0.8	4.6	2.2	2 (0.3)	0.845 (0.804–0.880)
	Cognitive demands	0.74	0.63	0.71	63.9	18.7	57.0	18.4	0.3	1.1	1.1	0.9	2.2	3 (0.4)	0.778 (0.721–0.828)
	Emotional demands	0.87	0.73	0.76	40.7	24.3	54.9	20.8	5.7	0.4	1.5	0.4	2.2	2 (0.3)	0.783 (0.727–0.831)
	Demands for hiding emotions	0.57	0.60	0.62	50.6	20.8	39.7	23.3	1.5	0.9	7.0	0.8	2.3	4 (0.5)	0.719 (0.644–0.784)
Work organisation and job contents	Influence	0.73	0.53	0.68	49.8	21.2	47.2	19.0	1.6	0.5	1.2	0.5	2.2	3 (0.4)	0.629 (0.531–0.712)
	Possibilities for development	0.77	0.71	0.73	65.9	17.6	68.7	17.0	0.4	2.3	0.3	4.4	2.6	2 (0.3)	0.810 (0.761–0.852)
	Variation	0.50	0.23	0.26	60.4	21.4	50.4	19.8	2.0	4.2	2.7	1.1	2.2	3 (0.4)	0.474 (0.324–0.598)
	Meaning of work	0.74	0.70	0.70	73.8	15.8	75.9	17.7	0.1	7.3	0.1	15.6	2.8	2 (0.3)	0.779 (0.745–0.844)
	Commitment to the workplace	0.76	0.61	0.71	60.9	20.4	69.5	16.4	0.7	2.2	0.1	4.7	2.2	2 (0.3)	0.521 (0.307–0.628)
Interpersonal relations and leadership	Predictability	0.74	0.50	0.62	57.7	20.9	58.6	19.5	1.5	4.2	1.2	3.0	2.3	2 (0.3)	0.736 (0.660–0.799)
	Recognition	0.83	0.67	0.76	66.2	19.9	66.9	19.2	0.9	5.8	0.4	4.4	2.8	2 (0.3)	0.807 (0.755–0.851)
	Role clarity	0.78	0.72	0.73	73.5	16.4	60.2	14.7	0.0	7.5	0.3	0.9	2.7	2 (0.3)	0.777 (0.717–0.828)
	Role conflicts	0.67	0.70	0.67	42.0	16.6	44.2	19.0	1.3	0.2	1.8	0.5	2.6	3 (0.4)	0.785 (0.729–0.833)
	Quality of leadership	0.89	0.90	0.88	55.3	21.1	64.6	21.5	1.2	1.9	0.3	10.7	2.0	167^a^ (22.4)	0.926 (0.903–0.945)
	Social support from colleagues	0.70	0.65	0.74	57.3	19.7	59.6	21.9	1.1	1.9	0.3	12.8	2.0	5 (0.7)	0.748 (0.748–0.808)
	Social support from supervisors	0.79	0.84	0.82	61.6	22.4	68.4	19.2	0.9	4.4	0.8	3.1	2.7	165^b^ (22.1)	0.834 (0.780–0.878)
	Social community at work	0.85	0.81	0.77	78.7	18.9	59.3	20.3	0.2	24.4	1.9	2.6	2.6	3 (0.4)	0.832 (0.787–0.870)
Work-individual interface	Job insecurity	0.77	0.77	0.79	23.7	20.8	43.9	26.1	19.0	0.5	6.9	2.0	2.3	2 (0.3)	0.835 (0.793–0.872)
	Job satisfaction	0.82	0.72	0.80	65.3	18.2	62.5	16.0	0.7	5.1	0.3	3.0	2.8	3 (0.4)	0.864 (0.826–0.897)
	Work-family conflict	0.80	0.84	0.85	33.5	24.3	40.0	26.7	9.7	1.2	9.3	4.0	2.9	3 (0.4)	0.905 (0.880–0.927)
	Family-work conflict	0.79	0.76	0.88	7.6	15.3	10.7	16.9	74.6	0.2	65.4	0.4	2.9	2 (0.4)	0.792 (0.751–0.842)
Values at the workplace	Mutual trust between employees	0.77	0.66	0.65	68.6	16.9	69.0	16.6	0.0	5.6	0.1	4.7	3.2	10 (1.3)	0.752 (0.685–0.809)
	Trust regarding management	0.80	0.60	0.65	67.0	17.7	62.8	18.2	0.2	3.9	0.7	3.7	2.5	7 (0.9)	0.785 (0.729–0.834)
	Justice	0.83	0.81	0.83	59.2	17.7	61.8	18.3	0.4	1.6	0.4	3.0	2.6	7 (0.9)	0.878 (0.846–0.906)
	Social inclusiveness	0.63	0.65	0.64	67.5	16.3	59.0	20.7	0.1	3.8	0.7	2.2	2.8	8 (1.1)	0.685 (0.601–0.758)
Health and well-being	General health perception	-	-	-	66.0	20.9	58.3	22.8	0.8	14.8	1.3	9.8	1.2	0 (0)	0.820 (0.753–0.869)
	Burnout	0.83	0.91	0.94	34.1	18.2	32.9	22.5	1.7	0.2	10.9	0.3	0.6	1 (0.1)	0.938 (0.922–0.952)
	Stress	0.81	0.83	0.87	26.7	17.7	43.9	22.3	5.2	0.1	4.0	1.5	0.6	1 (0.1)	0.904 (0.879–0.925)
	Sleeping troubles	0.86	0.88	0.93	21.3	19.0	38.7	21.6	17.4	0.0	5.4	0.3	0.6	2 (0.3)	0.930 (0.912–0.946)
	Depressive symptoms	0.76	0.77	0.82	21.0	16.5	32.9	22.5	10.3	0.0	10.9	0.3	0.7	1 (0.1)	0.862 (0.826–0.893)
	Somatic stress	0.68	0.70	0.78	17.8	16.0	26.9	18.9	16.6	0.0	12.2	0.3	0.6	1 (0.1)	0.843 (0.802–0.878)
	Cognitive stress	0.83	0.84	0.88	17.8	15.7	31.8	18.8	18.6	0.0	5.9	0.1	0.7	1 (0.1)	0.915 (0.893–0.934)
	Self-efficacy	0.80	0.80	0.89	67.5	16.0	66.1	17.9	0.0	1.8	0.1	2.3	1.3	1 (0.1)	0.890 (0.862–0.914)
Offensive behaviour	Sexual harassment	-	-	-	2.9%	-	0.6%	-	97.0	0.1	98.1	0.1	3.3	7 (0.9)	0.655 (0.526–0.749)
	Threats of violence	-	-	-	7.8%	-	1.5%	-	92.2	0.3	95.1	0.1	3.2	8 (1.1)	0.909 (0.875–0.934)
	Physical violence	-	-	-	3.9%	-	0.2%	-	96.1	0.0	99.2	0.8	3.3	8 (1.1)	0.888 (0.871–0.903)
	Bullying	-	-	-	8.3%	-	1.0%	-	91.7	0.5	96.7	0.1	2.5	8 (1.1)	0.562 (0.399–0.681)
	Unpleasant teasing	-	-	-	8.3%	-	5.2%	-	91.7	0.3	82.5	0.3	3.2	7 (0.9)	0.813 (0.743–0.864)
	Conflicts and quarrels	-	-	-	51.2%	-	5.8%	-	48.8	1.3	79.9	0.4	2.5	7 (0.9)	0.683 (0.564–0.769)
	Gossip and slander	-	-	-	38.9%	-	5.3%	-	61.1	3.5	83.6	0.8	2.6	7 (0.9)	0.658 (0.531–0.751)

^a^ Most cases are “not applicable” rather than there being “no answers” from participants. The data results of the “non-answers” and not applicable are the following for the two scales: *Quality of leadership* [no answers *n* = 10; not applicable *n* = 157] and *Social support from supervisors* [no answers *n* = 9; not applicable *n* = 156]

For 29 of the 41 scales, Cronbach’s alpha was generally above the conventional threshold of 0.70, nine scales ranged between 0.60 and 0.70, and three scales had a reliability of less than 0.60 (*Influence at work*, *Variation* and *Predictability*). Test-retest reliability was assessed by examining the correlation of the scale score in the baseline long version of the COPSOQ II questionnaire with the COPSOQ II questionnaire scale score completed 2 weeks after the baseline assessment. According to the adopted criteria for the interpretation of the magnitude of the ICC (> 0.70), this analysis indicated an acceptable reliability for 33 out of 41 scales. For the eight scales where we had ICC values of less than 0.70, five of them had very close values and three were indicative of poor reliability.

The average scores and standard deviations showed similar results to the original Danish study [16]. However, the average scores showed moderate differences in eight scales [Demand for hiding emotions (Portugal = 39.7, Denmark = 50.6), Social support from supervisors (Portugal = 68.4, Denmark = 61.6), Social community at work (Portugal = 59.3, Denmark = 78.7), Stress (Portugal = 43.9, Denmark = 26.7), Sleeping troubles (Portugal = 38.7, Denmark = 21.3), Depressive symptoms (Portugal = 32.9, Denmark = 21.0), Somatic stress (Portugal = 26.9, Denmark = 17.8) and Cognitive stress (Portugal = 31.8, Denmark = 17.8)] and very significant differences in three scales [Job insecurity (Portugal = 43.9, Denmark = 23.7), Conflicts and quarrels (Portugal = 5.8%, Denmark = 51.2) and Gossip and slander (Portugal = 5.3%, Denmark = 38.9)]. These verified differences are positive and negative, depending on each case.

Most of the scales had low floor and ceiling effects, except *Family–work conflict,* which had a high floor effect (65.4%).

For 39 of the 41 scales in the long questionnaire, the percentage of missing values was less than 1.3% (0.1–1.3%). Two scales had high values [*Quality of leadership* (22.4%) and *Social support from supervisors* (22.1%)] although most cases are not applicable rather than there being no answers from participants.

An exploratory factor analysis was conducted considering the seven dimensions of the long version of the COPSOQ II, and the results are summarized in Tables 5, 6, 7, 8, 9, 10, 11.

**Table 5:**
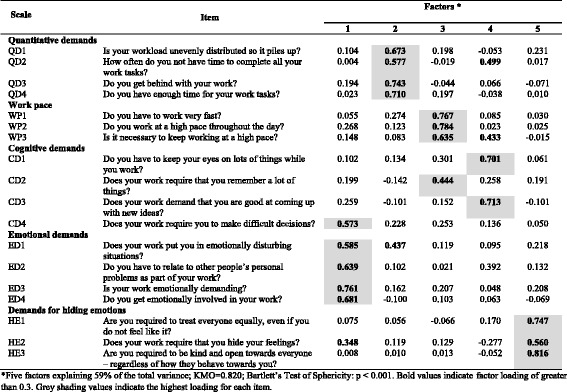
Exploratory factor analysis of items in the *Demands at work* dimension (*n* = 700) of COPSOQ II (long version): loadings for each factor and each item in the scale after varimax rotation and factor extraction using principal components

*Five factors explaining 59% of the total variance; KMO = 0.820; Bartlett’s Test of Sphericity: *p* < 0.001. Bold values indicate factor loading of greater than 0.3. Grey shading values indicate the highest loading for each item

**Table 6:**
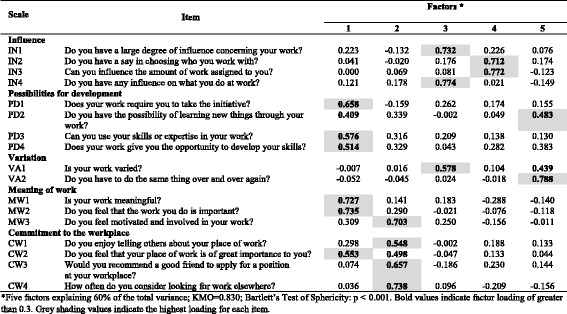
Exploratory factor analysis of items in the *Work organisation and job contents* dimension (*n* = 699) of COPSOQ II (long version): loadings for each factor and each item in the scale after a varimax rotation and factor extraction using principal components

*Five factors explaining 60% of the total variance; KMO = 0.830; Bartlett’s Test of Sphericity: *p* < 0.001. Bold values indicate factor loading of greater than 0.3. Grey shading values indicate the highest loading for each item

**Table 7:**
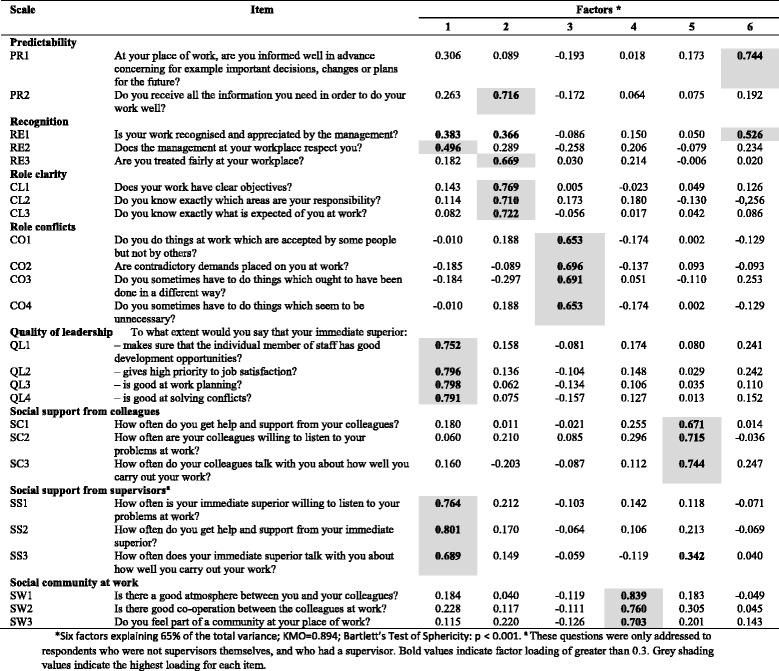
Exploratory factor analysis of items in the *Interpersonal relations and leadership* dimension (*n* = 516) of COPSOQ II (long version): loadings for each factor and each item in the scale after a varimax rotation and factor extraction using principal components

*Six factors explaining 65% of the total variance; KMO = 0.894; Bartlett’s Test of Sphericity: *p* < 0.001. ^**a**^ These questions were only addressed to respondents who were not supervisors themselves, and who had a supervisor. Bold values indicate factor loading of greater than 0.3. Grey shading values indicate the highest loading for each item

**Table 8:**
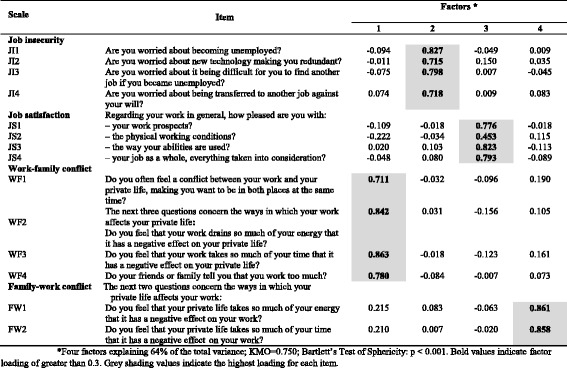
Exploratory factor analysis of items in the *Work-individual interface* dimension (*n* = 704) of COPSOQ II (long version): loadings for each factor and each item in the scale after a varimax rotation and factor extraction using principal components

*Four factors explaining 64% of the total variance; KMO = 0.750; Bartlett’s Test of Sphericity: *p* < 0.001. Bold values indicate factor loading of greater than 0.3. Grey shading values indicate the highest loading for each item

**Table 9:**
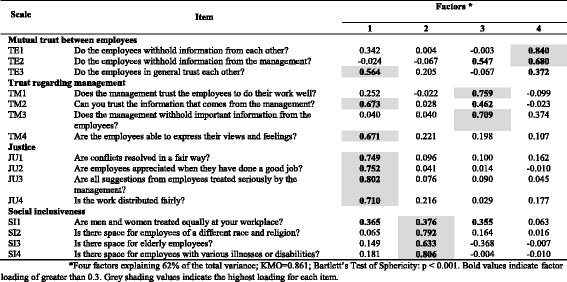
Exploratory factor analysis of items in the *Values at the workplace* dimension (*n* = 683) of COPSOQ II (long version): loadings for each factor and each item in the scale after a varimax rotation and factor extraction using principal components

*Four factors explaining 62% of the total variance; KMO = 0.861; Bartlett’s Test of Sphericity: *p* < 0.001. Bold values indicate factor loading of greater than 0.3. Grey shading values indicate the highest loading for each item

**Table 10:**
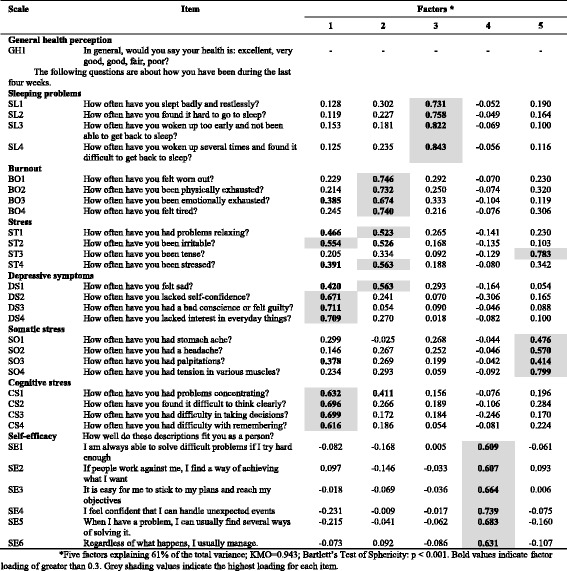
Exploratory factor analysis of items in the *Health and well-being* dimension (*n* = 694) of COPSOQ II (long version): loadings for each factor and each item in the scale after a varimax rotation and factor extraction using principal components

*Five factors explaining 61% of the total variance; KMO = 0.943; Bartlett’s Test of Sphericity: *p* < 0.001. Bold values indicate factor loading of greater than 0.3. Grey shading values indicate the highest loading for each item

**Table 11:**
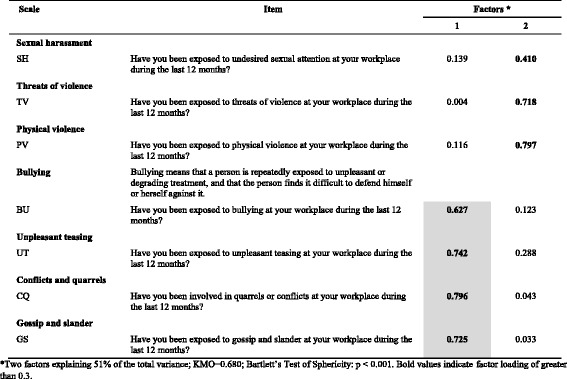
Exploratory factor analysis of items in the *Offensive behaviour* dimension (*n* = 729) of COPSOQ II (long version): loadings for each factor and each item in the scale after a varimax rotation and factor extraction using principal components

*Two factors explaining 51% of the total variance; KMO = 0.680; Bartlett’s Test of Sphericity: *p* < 0.001. Bold values indicate factor loading of greater than 0.3

In the *Demands at work* dimension, the results support the scales (*Quantitative demands, Work pace, Emotional demands* and *Demands for hiding emotions*). However, items in the scale of *Cognitive demands* have the highest loadings on three different factors, indicating that the construct validity of this scale is not supported (Table 5).

In the *Work organisation and Job Contents* dimension, the results support the *Commitment to the workplace* scale. Items in the *Influence* scale are split into two factors (one factor concerning influence in general and concerning what you do, and one factor concerning influence on who you work with and the amount of work). In the scale concerning *Possibilities for development*, one item loads on factor 5 rather than factor 1. In the *Variation* scale, one item loads on factor 5 while the other item loads highest on factor 3, together with two items concerning *Influence*. In the *Meaning of work* scale, one item loads highest on factor 2, together with the items on *Commitment to the workplace* (Table 6).

In the *Interpersonal relations and leadership* dimension (Table 7), the results support the *Role clarity, Role conflict, Social support from colleagues* and *Social community at work* scales. Two scales (*Quality of leadership* and *Social support from supervisors*) load on the same factor. Two other scales (*Predictability* and *Recognition*) load on several factors.

In the *Work-individual interface* dimension, the results support the hypothesised scale structure (Table 8).

In the *Values at the workplace* dimension, the results support the hypothesised scale structure for two scales (*Justice* and *Social inclusiveness*), while the other two scales are split between several factors (Table 9).

In the *Health and well-being* dimension, the results support the hypothesised scale structure for five scales (*Sleeping problems, Burnout, Somatic stress, Cognitive stress* and *Self-efficacy*). The stress scale is split into several factors. In the *Depressive symptoms* scale, DS1 loads strongest on factor 2 (Table 10).

In the *Offensive behaviour* dimension (Table 11), the results support the *Bullying, Unpleasant teasing, Conflict and quarrels* and *Gossip and slander* scales. The other three scales load on factor 2 rather than factor 1.

The results of the exploratory factor analysis showed that, from the 41 total scales, 27 support the hypothesised scale structure while the factor results differ from the scale structure for 14 scales (Cognitive demands, Influence, Possibilities for development, Variation, Meaning of work, Predictability, Recognition, Mutual trust between employees, Trust between management, Stress, Depressive symptoms, Sexual harassment, Threats of violence and Physical violence).

## Discussion

This paper described the Portuguese validation of the long version of COPSOQ II using rigorous methodology based on both psychometric and conceptual criteria.

In general, a Cronbach’s alpha of the COPSOQ scales (test and retest) indicated acceptable reliability (0.7). Furthermore, the fact that Cronbach’s alpha is influenced by the number of items in the scale explains the findings of lower values of alphas.

The test-retest reliability results indicate that most of the scales showed good temporal stability and reliability in the considered time interval. However, there were eight scales that showed ICC values below 0.7 (*Influence, Variation, Commitment to the workplace, Social inclusiveness, Sexual harassment, Bullying, Conflicts and quarrels and Gossip and slander*). Out of these eight four belonged to the offensive behaviour dimension, three to belonged to the Work organisation and job contents and the remaining one to the Values at workplace dimension.

The three scales concerning the *Variation*, *Commitment to the workplace and Bullying* showed poor ICC values. The reason for the poor test-retest reliability should be evaluated in future studies.

The test-retest design showed a good reliability for most of the scales, namely where Cronbach’s alpha was low, as reported in a previous study by Thorsen and Bjorner [67]. These authors examined the reliability of the COPSOQ work environment questionnaire and have concluded that the test-retest design and intraclass correlation appears to be more appropriate than Cronbach’s alpha for assessing the reliability of COPSOQ’s psychosocial work environment scales.

Thorsen and Bjorner [67] specified assumptions for 26 COPSOQ scales, eight of each were assumed to exhibit a reflective model (internal consistency) and 18 were assumed to exhibit a formative model.

The exploratory factor analysis findings assumed that from the 41 total scales, 27 are based on a reflective model of effect indicators, in which all of the items are a manifestation of the same underlying construct [46, 68, 69]. The remaining 14 scales did not show a clear factor in the exploratory factor analysis. Out of these, three (Meaning of work, Stress and Depressive symptoms) cannot be assumed to exhibit the formative model, since they had previously been assumed to exhibit a reflective measurement model, as reported by Thorsen and Bjorner [67]. Future studies should evaluate these three scales in greater depth.

The remaining 11 scales assumed to exhibit a formative model in which items are combined due to their hypothesised common effect rather than their common cause. High inter-item correlation is not a necessary criterion of construct validity and these do not need to be correlated [46, 67, 70, 71].

Following this line of thinking, as Thorsen and Bjorner [67] also state, Cronbach’s alpha might not be a good measure of reliability for these scales because it might underestimate true reliability. In this circumstance, the internal consistency is not considered relevant for items that form a formative model [46, 70–74].

In accordance with these findings, the authors Bjorner and Pejtersen [75] argue that the traditional psychometric techniques (e.g. factor analysis and reliability through Cronbach’s alpha) may not be appropriate for some COPSOQ II scales for which the items are combined based on a hypothesised common effect rather than a hypothesised common cause.

As quoted in their work [75] “*Bollen pointed out that not all questionnaires scales can be conceived as consisting of effect indicator items, being that some items must be seen as causes of the latent construct rather than effects”* [70, 71].

These insights can help to explain the apparently “inconsistent” findings that were reported in some of the results of the exploratory factor analysis.

The average scores and standard deviations showed similar results to the original Danish study, except for 11 scales, which may be explained by the context of unstable labour markets and the significant increase in employees’ feeling of job insecurity (e.g. fear of being hampered in the performance of their function or in their career development and even of losing their job) and the resulting negative impact on employees’ health and well-being. As for the floor and ceiling effect, we observed similar results to the original Danish study. The *Family-work conflict* scale showed a high floor effect (65.4%) and a very low mean value (10.7). In accordance with the original authors, this result also indicates that private life is not interfering with work in general.

As for the missing items, in 39 out of the total of 41 scales, the missing items are less than 1.3%. A higher proportion of missing values observed in two scales (*Quality of leadership* and *Social support from supervisors*) should be interpreted cautiously due to the fact that most cases are “not applicable” questions rather than “no answers” from the participants.

The Portuguese COPSOQ II had a moderate response rate of 60.6% for the baseline test (*n* = 745) and a good follow-up rate of 59.5% for the retest (*N* = 394).

Several strengths of this study need to be mentioned. Firstly, the inclusion of international statistical standards enables reliable and comparable national, European and international statistics. In line with this, validation of the long version of COPSOQ II, maintaining its full content and structure, also enables statistics comparable to those of other countries.

Secondly, the adoption of COSMIN methodology, internationally widely accepted recommendations for the assessment of psychometric characteristics, is aimed at ensuring the quality of results. Thirdly, the inclusion of various sectors of economic activity, taking into consideration workers at different hierarchical levels and in different functions in each company, ensured greater confidence in the results.

There were some limitations to the study. Firstly, the study sample in the *Wholesale and retail trade* and *Manufacturing* sectors of economic activity should be improved. Secondly, the online survey data collection had lower response rates than the paper-based ones. Thirdly, the current economic crisis could have an impact on the answers that people give to some of the questions.

## Conclusion

Most scales in the Portuguese long version of the COPSOQ II were found to be valid and reliable for the evaluation and study of the implications of psychosocial work factors for the health and well-being of workers. Three scales need further evaluation since the hypothesized factor structure was not supported (Meaning of work, Stress, and Depressive symptoms) while three other scales should be further evaluated due to low reliability in test-retest analyses (Variation, Commitment to the workplace, and Bullying).

The Framework Directive (89/391/EEC) confers a central place in risk assessment to preventive approaches and highlights the use of valid and reliable methods in order to identify all types of risk factors in organisations, with psychosocial risk management being the employers’ responsibility. This line of approach establishes the importance of integrated prevention, taking an increasing number of risk factors into consideration and including all aspects of psychosocial risks (e.g. demands at work, work-individual interface, work organisation and job contents, offensive behaviour, etc.). This tool is intended to be a resource for researchers and professionals in Portuguese organisations for the prevention and promotion of health and well-being in the labour context and also to promote the development of a national culture of prevention, in particular as regards psychosocial risk factors.

In future research, gradual use of the COPSOQ in various economic activities will lead to a broader database, thereby allowing researchers and professionals to adjust validation analyses (in particular the scales that indicated less satisfactory results), establish comparisons between companies and advance in the development of Portuguese standards.

## Additional file

Additional file 1: Portuguese Classification of Economic Activities (CAE) – Revision 3 (CAE – Rev. 3) according to Pordata (2013). (DOCX 16 kb)

## Abbreviations

BGW: Institute for statutory accident insurance and prevention in the health and welfare services
CAE – Rev. 3: Portuguese classification of economic activities – revision 3
CIDES: Department of health information and decision sciences
CINTESIS: Centre for research in health technologies and information systems and information and decision sciences department
COPSOQ II: Copenhagen Psychosocial Questionnaire II
COSMIN: COnsensus-based standards for the selection of health measurement instruments
CUF: Companhia União Fabril
Cvcare: Center of excellence for epidemiology and health service research for healthcare professionals
EUROSTAT: Statistical office of the european communities
FFAW GmbH: Freiburg research centre for occupational sciences
GPR: Principles of prevention and rehabilitation department
ICC: Intraclass correlation coefficient
ISCO: International standard classification of occupations
ISIC – Rev. 4: International standard classification of activities – revision 4
IVDP: Institute for health services research in dermatology and nursing
KMO: Kaiser-Meyer-Olkin
LAETA: Associated laboratory for energy, transport and aeronautics
NACE – Rev. 2: Classification of economic activities in the european union – revision 2
NOPain: National observatory of pain
OECD: Organisation for Economic Co-operation and Development
OSH: Occupational Safety and Health
PORDATA: The data base of contemporary Portugal
SD: Standard deviation
SPSS: Statistical package for the social sciences
