# Supplementary material for: The Portuguese long version of the Copenhagen Psychosocial Questionnaire II (COPSOQ II) – a validation study
Source: J Occup Med Toxicol. 2017 Aug 9;12:24. doi: 10.1186/s12995-017-0170-9 (PMC5550997; doi:10.1186/s12995-017-0170-9)
Supplement: Additional file 1: — Portuguese Classification of Economic Activities (CAE) – Revision 3 (CAE – Rev. 3) according to Pordata (2013). (DOCX 16 kb) [file 12995_2017_170_MOESM1_ESM.docx]

**Additional file 1** Portuguese Classification of Economic Activities (CAE) – Revision 3 (CAE – Rev. 3) according to Pordata (2013)

| Section | Description | Pordata 2013 | | Study sample | |
| --- | --- | --- | --- | --- | --- |
|  |  | N | % ^1^ | n | % |
| A | Agriculture, forestry and fishing | 160,959 | 4.6 | - | - |
| B | Mining and quarrying | 9,628 | 0.3 | - | - |
| C | Manufacturing | 637,427 | 18.3 | 53 | 7.1 |
| D | Electricity, gas, steam and air conditioning supply | 38,858 | 1.1 | - | - |
| F | Construction | 307,907 | 8.8 | 56 | 7.5 |
| G | Wholesale and retail trade; repair of motor vehicles and motorcycles | 723,488 | 20.8 | 43 | 5.8 |
| H | Transportation and storage | 147,757 | 4.2 | - | - |
| I | Accommodation and food service activities | 265,694 | 7.6 | - | - |
| K | Financial and insurance activities | 107,213 | 3.1 | 21 | 2.8 |
| L | Real estate activities | 45,299 | 1.3 | - | - |
| P | Education | 91,749 | 2.6 | 161 | 21.6 |
| Q | Human health and social work activities | 150,020 | 4.3 | 267 | 35.8 |
| E,J,M,N,O,R,S,T,U | Other sectors^2^ | 794,732 | 22.8 | 144 | 19.3 |
|  | Total | 3,480,731 | 100 | 745 | 100 |

^1^ The respective percentage of the total of organisations by economic activity.

^2^ The sectors E (water supply, sewerage, waste management and remediation activities), J (information and communication), M (professional, scientific and technical activities), N (administrative and support service activities), O (public administration and defence; compulsory social security), R (arts, entertainment and recreation), S (other service activities), T (activities of households as employers; undifferentiated goods- and services- producing activities of households for own use) and U (activities of extraterritorial organisations and bodies) designated as “Other sectors” according to Pordata which correspond to NACE – Rev. 2 and ISIC-REV classification.
